# Sidearm Deliveries Are Associated With Lower Shoulder and Elbow Injury Burden and a Favorable Performance Profile in Major League Baseball: A Cohort Study of 927 Pitchers (2020-2025)

**DOI:** 10.1177/23259671261470537

**Published:** 2026-08-26

**Authors:** Morgan R. Dillon, Michael A. Mastroianni, Nicholas Frappa, Joseph Manzi, Samuel I. Fuller, William Curtis, Frank J. Alexander, Robert Ablove, Peter N. Chalmers, Christopher S. Ahmad

**Affiliations:** * Jacobs School of Medicine and Biomedical Sciences, Buffalo, New York, USA; † Department of Orthopedic Surgery, Columbia University Irving Medical Center–New York Presbyterian Hospital, New York, New York, USA; ‡ Department of Orthopaedics, Lenox Hill Hospital, New York, New York, USA; § University at Buffalo Department of Orthopaedics and Sports Medicine, Buffalo, New York, USA; ‖ Department of Orthopaedic Surgery, University of Utah, Salt Lake City, Utah, USA; Investigation performed at Columbia University, New York City, NY, USA

**Keywords:** pitching, Major League Baseball, arm slot, shoulder, elbow, Statcast

## Abstract

**Background:**

Pitching-related shoulder and elbow injuries continue to rise in baseball despite workload restrictions and biomechanics-informed coaching.

**Purpose:**

To evaluate associations between arm slot and shoulder/elbow injured list (IL) time-loss burden and performance in Major League Baseball (MLB) pitchers.

**Study Design:**

Cohort study; Level of evidence, 3.

**Methods:**

Publicly available data were aggregated for 927 MLB pitchers who threw ≥1000 pitches during 2020 to 2025 and had arm angle data available from the MLB Hawk-Eye tracking system. Arm slot was defined as the throwing-arm angle relative to horizontal at ball release and averaged per pitcher (mean ± SD, 39.0°± 10.9°). Based on the distribution of arm slot values in the cohort, pitchers were categorized as sidearm (<33.5°; n = 255), three-quarters (33.5°-44.4°; n = 373), or overhand (>44.4°; n = 299). Primary outcomes were cumulative shoulder+elbow, shoulder, and elbow IL days accrued during the 2020 to 2025 study period. Performance outcomes included run prevention metrics, FanGraphs "plus" metrics (Stuff+ reflects pitch quality indexed to league average), strikeout and walk rates, hard-hit percentage (frequency of high-exit-velocity contact), and expected fielding independent pitching (xFIP; pitcher-controlled run prevention estimate). Group comparisons used 1-way analysis of variance with Tukey testing. Continuous associations with performance metrics reaching group-level significance were assessed with linear regression. Injury burden was modeled using negative binomial regression with log (total pitches) and percentage of games started as covariates; the primary analysis additionally adjusted for Stuff+ and 4-seam fastball velocity to test for independent associations after accounting for established risk factors.

**Results:**

Pitchers accumulated 86,412 combined IL days (25,710 shoulder; 60,702 elbow). Shoulder injury burden increased linearly with more overhand arm slots, whereas combined and elbow burden were nonlinear, peaking within the overhand group (45°-57°). Per 1 SD (10.9°) toward a more overhand delivery, injury burden increased for combined (incidence rate ratio [IRR] = 1.09; *P* = .01), shoulder (IRR = 1.11; *P* = .002), and elbow (IRR = 1.08; *P* = .03). After adjustment for Stuff+ and 4-seam velocity, arm slot remained independently associated with higher injury burden (IRR = 1.12-1.14; all *P* < .001). Performance profiles favored sidearm deliveries: per 1-SD more overhand slot, Stuff+ decreased (β = −1.09; *P* < .001), while xFIP (β = 0.05; *P* = .02) and hard-hit percentage (β = 0.50; *P* < .001) increased. Sidearm pitchers used fewer 4-seam fastballs/cutters and more sinkers/sweepers.

**Conclusion:**

In this MLB cohort, sidearm deliveries were associated with lower shoulder/elbow time-loss burden, superior pitch quality, and lower hard-hit rates. Shoulder injury burden increased linearly with more overhand deliveries, whereas elbow burden followed a nonlinear pattern that peaked within the overhand range. These associations were independent of workload, velocity, and pitch quality. Arm slot may represent a clinically relevant marker of pitcher injury risk that warrants prospective study.

Pitching-related shoulder and elbow injuries remain pervasive across all levels of baseball, from youth and amateur competition to the professional ranks, and continue to impose substantial clinical, developmental, and performance-related costs.^8,20,24,28^ Despite decades of research, advances in sports medicine, and widespread adoption of workload monitoring and restrictions, injury rates continue to rise. This persistent injury burden exists alongside a fundamental tension in modern baseball: contemporary coaching and player development, increasingly guided by biomechanical feedback and data analytics, are explicitly designed to maximize velocity and ball movement.^24^ These traits are central to performance success, but they are also closely tied to increased mechanical stress and injury risk.^2,41^ As pitchers at every level continue to throw harder and with more movement than ever before, the challenge is no longer whether these performance traits will be pursued, but whether injury risk can be reduced without sacrificing them. This has created an urgent need to identify mechanically relevant risk factors that influence injury risk beyond simply reflecting how hard or how well a pitcher throws.

One of the most visible yet poorly understood of these factors is arm angle, or pitching slot. Pitchers are commonly categorized as overhand, three-quarter, or sidearm throwers according to whether the arm is positioned nearer vertical, intermediate, or nearer horizontal at ball release.^13^ Despite its routine emphasis in coaching and player evaluation, existing biomechanical literature regarding arm slot is relatively sparse and highly heterogeneous. Historical analysis has largely been confined to the laboratory setting, utilizing inconsistent definitions that rely on proxies or individual kinematic contributors, such as contralateral trunk tilt or forearm orientation.^3,7,32,36,37,42^ In contrast, more recent analyses define arm slot directly as the angle of the shoulder-to-ball vector at release (Figure 1), capturing the overall arm orientation produced by trunk tilt, shoulder abduction, and elbow flexion.^12,13,25^ Consequently, findings regarding kinetics and injury mechanisms remain contradictory. While some studies link overhand mechanics to increased ball velocity and elbow joint loads,^12,25,33,37,42^ others have found the opposite, associating sidearm delivery with increased medial elbow torque and pathology.^3,4,36^ Recent analyses suggest a potential trade-off: sidearm arm slots may reduce elbow varus torque, a well-established mechanical correlate of ulnar collateral ligament (UCL) injury,^5,16,18^ yet may concurrently increase elbow flexion torque.^13,25^ Because elbow flexion torque is primarily generated by the biceps brachii, this loading pattern has been implicated in injury mechanisms involving the superior labrum and proximal biceps tendon in overhead throwers.^16,25^ Given the clear effects of arm slot and its surrogates on ball velocity and throwing-arm kinetics, most of the literature has recommended a three-quarter delivery as the safest compromise.^13,25^ Yet, a 2023 analysis of professional pitchers by Escamilla et al^14^ found higher peak shoulder and elbow forces in pitchers with a three-quarter arm slot, challenging the assumption that neutrality equates to safety.

**Figure 1.**
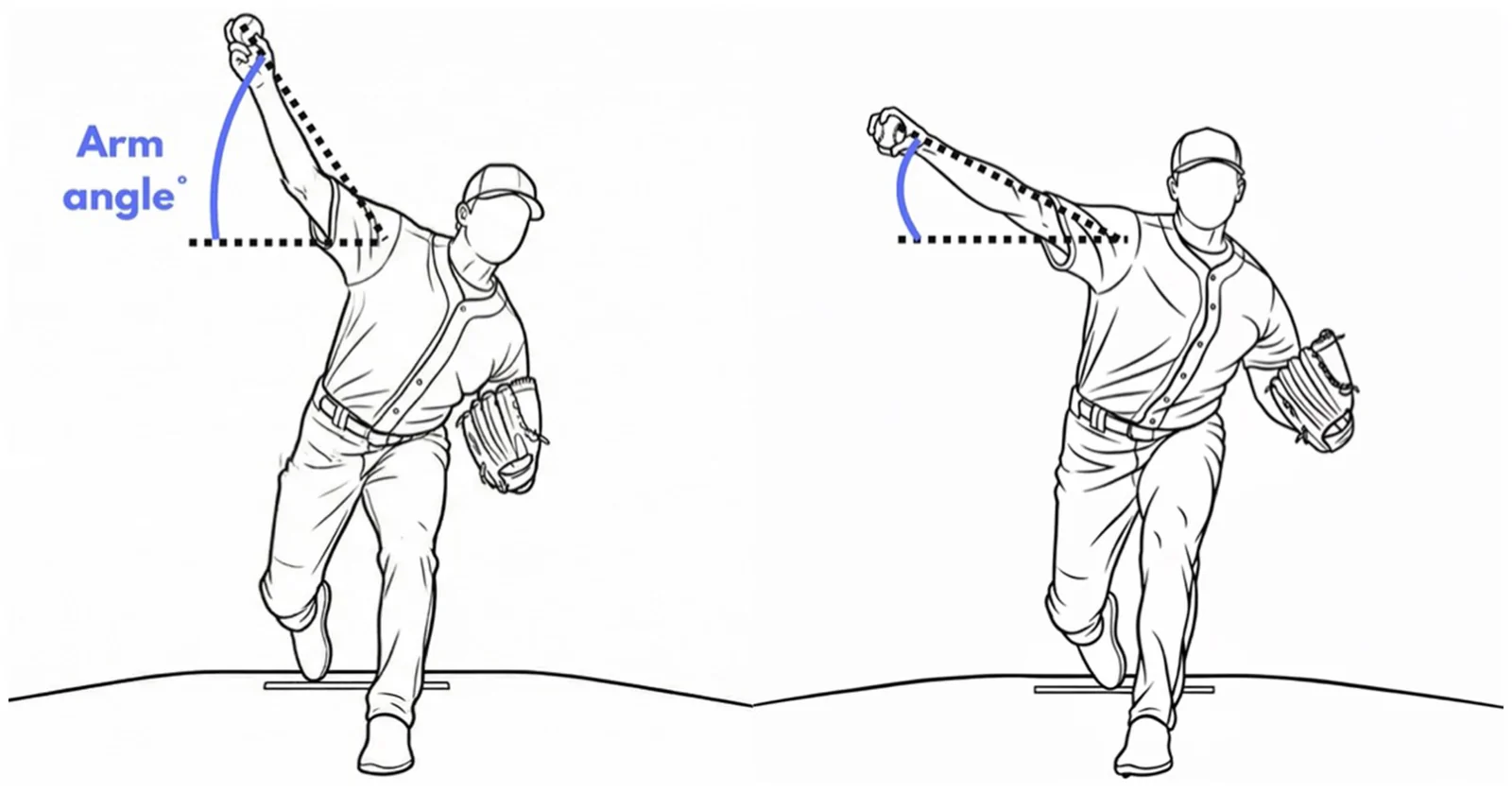
Illustration of arm angle/slot measurement. Arm slot was measured at ball release as the angle between the throwing arm and the horizontal plane. Higher arm slot values indicate a more overhand delivery (left), whereas lower values indicate a more sidearm delivery (right). Image adapted with permission from Mastroianni MA, Dillon MR, Frappa N, et al. Pitch-tracking risk factors and warning signs for ulnar collateral ligament injuries in Major League Baseball pitchers. Am J Sports Med. 2026;54(3):694-704.

Beyond the laboratory, Major League Baseball (MLB) is one of the few settings where pitching kinematics can be studied at true scale and linked to clinically meaningful outcomes. Since Statcast was implemented leaguewide in 2015, every pitch has been captured with pitch-level tracking data—including velocity, spin, movement, and release coordinates—creating a massive longitudinal data set capable of connecting mechanical signatures to performance and injury burden.^35,40^ A growing body of Statcast-based work has leveraged this infrastructure to evaluate injury risk, most often focusing on UCL outcomes, and has repeatedly implicated high-velocity, high-spin, and higher-performing pitchers as having increased injury risk.^26,29,31,39,44^ Yet most of these studies lacked a biomechanically defined arm slot variable, which has only become available with the integration of the Hawk-Eye tracking system. Hawk-Eye was implemented leaguewide beginning in 2020, but arm angle data were not publicly reported until 2024, at which point historical data from 2020 onward became accessible. In the absence of a direct measure, prior studies in MLB pitchers relied on horizontal and vertical release positions, measured as the ball's location at release relative to the center of the mound and its height from the mound, respectively, as surrogates for arm slot. However, release position is influenced by multiple factors that can shift the ball's location at release without reflecting meaningful changes in arm slot, including player height, arm length, stride direction, and mound positioning.^27,29^ As a result, proxy-based studies have produced inconsistent conclusions, with more medial release points (often interpreted as a higher slot) linked to increased UCL injury risk in some analyses,^31,44^ the opposite association in others,^39^ and no association in others.^26,29^

Beyond injury, arm slot may also shape performance. Prior biomechanical studies have shown that arm slot and related trunk/arm positioning influence pitch velocity and ball movement, suggesting that different arm slot strategies may produce distinct pitch-quality profiles.^6^ In MLB, modern pitching evaluation increasingly relies on advanced analytics that quantify underlying pitch characteristics, contact quality, and run prevention skill.^34^ Although these measures are now widely used to characterize pitcher effectiveness,^29-31^ the extent to which they vary across the arm slot spectrum has not been well-defined at the league level.

With arm slot data now publicly reported, it is possible to evaluate this commonly discussed mechanical feature using directly measured MLB tracking data rather than release-point proxies or small laboratory cohorts. The purpose of this study was to determine whether arm slot is associated with clinically meaningful shoulder and elbow injury burden in MLB pitchers using a longitudinal leaguewide data set. A secondary aim was to examine whether performance profiles differ across the arm slot spectrum. We therefore tested whether arm slot was independently associated with shoulder and elbow injury burden after adjusting for fastball velocity and pitch quality (Stuff+), while also comparing advanced performance metrics across arm slot groups. We hypothesized that arm slot would be associated with distinct injury and performance profiles across the arm slot spectrum, independent of fastball velocity and pitch quality.

## Methods

### Study Design and Data Sources

This retrospective cohort study evaluated associations between pitcher-level arm slot, upper-extremity injury burden, and performance among MLB pitchers from 2020 through 2025. Player demographics were obtained from official MLB sources.^38^ Performance metrics were obtained from FanGraphs,^15^ and pitch-level tracking data was obtained from Baseball Savant (Statcast).^40^ These data were aggregated using Baseballr.^38^ Shoulder- and elbow-specific injured list (IL) placements were identified through Spotrac^43^ and Fangraphs^15^ injury filters and manually verified via targeted searches of publicly available team and media reports before inclusion.^23^ Consistent with prior MLB studies using publicly available injury data,^11,26,27,29,31^ this process verified anatomic site and throwing-arm laterality, and placements attributed to nonmusculoskeletal conditions such as lacerations or contusions were excluded. Because this study used publicly available data, it was determined to be not human participants research and was therefore exempt from institutional review board review under 45 CFR 46.102.

### Study Population

Pitchers were eligible for inclusion if they threw ≥1000 MLB pitches during the 2020 to 2025 study window, ensuring adequate exposure for stable pitcher-level estimates. Of 1785 pitchers who appeared in ≥1 MLB game during this period, 927 met the pitch-count threshold and had available arm slot data, comprising the final analytic cohort. A sensitivity analysis restricted to pitchers with ≥2000 pitches (n = 644) yielded similar findings, with the primary associations between arm slot and shoulder and elbow injury burden, as well as the principal performance findings, remaining consistent in direction and magnitude.

### Variables

Demographic variables including age, height (cm), weight (kg), body mass index (BMI; kg/m^2^), and handedness (right/left) were recorded. Career workload and role were characterized by total pitches thrown and percentage of games started (2020-2025).

#### Pitch Characteristics

Statcast pitch-level data from 2020 through 2025 were aggregated across the study window to calculate each pitcher's mean pitch usage percentage, velocity, and spin rate for 7 pitch types: 3 fastball variants (4-seam fastball, sinker, cutter) and 4 off-speed pitches (slider, sweeper, curveball, changeup).

#### Primary Exposure

Arm slot was obtained from Statcast and is defined as the angle between a line from the throwing shoulder to the ball at release and the horizontal plane, reported in degrees (Figure 1). This approach is consistent with prior biomechanical studies of arm slot, although some studies report the angle relative to the vertical rather than horizontal plane, which changes the numeric scale but not the underlying construct.^12,13,25^ Accordingly, absolute degree values should not be interpreted as directly interchangeable across studies using different reference planes or tracking systems. Within this study, arm slot was therefore interpreted relative to the cohort distribution, with pitchers categorized as more sidearm, three-quarters, or overhand relative to MLB peers. A pitcher-level mean was used because the goal of the study was to characterize habitual delivery style rather than season-specific or pitch-specific variation. This approach was supported by minimal within-pitcher variation across both time and pitch type. The mean season-to-season change in arm angle was –0.35°, and the mean directional difference between each pitcher's 2 most-thrown pitch types was 0.11°, indicating negligible systematic variation in either dimension.

The cohort mean for arm slot was 39.0°± 10.9°. Pitchers were categorized into 3 groups based on the cohort distribution, consistent with prior biomechanical analyses,^12,25^ with sidearm and overhand groups defined as values >0.5 SD below and above the mean, respectively:

Sidearm: <33.5° (mean − 0.5 SD; n = 255)
Three-quarters: 33.5°-44.4° (mean ± 0.5 SD; n = 373)
Overhand: >44.4° (mean + 0.5 SD; n = 299)

#### Outcome Measures

Upper extremity injury burden was quantified as cumulative IL days across the study period for 3 outcomes: combined shoulder and elbow injury burden (total: 86,412 days), shoulder alone (25,710 days), and elbow alone (60,702 days). Pitching performance was assessed using both advanced and traditional baseball metrics, which were summarized at the pitcher level across the study window by averaging each pitcher's seasonal values. Earned run average (ERA) reflects the number of runs a pitcher allows per 9 innings and represents observed run prevention influenced by defense, sequencing, and context. Expected fielding independent pitching (xFIP) estimates a pitcher's underlying run prevention skill by modeling outcomes primarily driven by the pitcher such as strikeouts, walks, and home runs, while minimizing the influence of defense and batted-ball variance.

Pitch-level performance scores were assessed using FanGraphs “plus” metrics.^34^ Stuff+ evaluates the intrinsic physical characteristics of a pitcher's arsenal, including velocity, movement, and spin reflecting the ability to generate difficult-to-hit pitch shapes independent of location.^34^ Location+ evaluates a pitcher's ability to place pitches in advantageous regions of the strike zone after accounting for pitch type and count context, independent of pitch velocity or movement.^34^ Pitching+ integrates pitch physical characteristics, location, batter handedness, and count context to estimate the overall quality of a pitcher.^34^ All “plus” metrics are scaled such that 100 represents league average, with higher values indicating better performance. Additional metrics included strikeout percentage, walk percentage, and swinging-strike percentage, which capture bat-missing ability and control, as well as hard-hit percentage, defined as the proportion of batted balls with an exit velocity ≥95 mph.

### Statistical Analysis

Demographic variables, pitch characteristics, and performance metrics were compared across arm slot groups using 1-way analysis of variance (ANOVA) with the Tukey honestly significant difference post hoc test for pairwise comparisons. Performance measures that reached group-level significance in ANOVA were analyzed as independent variables in linear regression models, with standardized arm slot (per 1 SD, 10.9°) as the predictor. Associations are reported as regression coefficients (β) with 95% CIs, representing the change in each metric per 1-SD increase in arm slot. Associations between continuous arm slot and injury burden were assessed using negative binomial generalized linear models, which account for overdispersion in count data. To account for differences in pitching exposure and role, models included the natural logarithm of total pitches thrown and percentage of appearances as a starting pitcher as covariates. Arm slot was standardized to its standard deviation (10.9°) for interpretability; results are reported as incidence rate ratios (IRRs) per 1-SD increase with 95% CIs. Nonlinearity was assessed by testing quadratic terms and visual inspection using restricted cubic spline fits. For outcomes demonstrating significant nonlinear relationships (*P* < .05 for the squared term), peak injury burden was calculated as –β_1_/(2β_2_) and converted to degrees. Univariable models examining arm slot without adjustment for velocity or pitch quality were conducted to characterize unadjusted associations. The primary hypothesis was then tested using multivariable models that additionally adjusted for Stuff+ and 4-seam fastball velocity to determine whether arm slot remained independently associated with injury burden after accounting for these established risk factors.^10,27,31^ Collinearity was assessed using variance inflation factors; all values were <2, indicating no multicollinearity concern. All analyses were performed using Python 3.13.5 with statsmodels. Statistical significance was defined as *P* < .05 (2-tailed).

## Results

### Cohort Characteristics

Demographic and performance characteristics stratified by arm slot are presented in Table 1. Height and BMI differed significantly across groups. Sidearm pitchers were taller than both three-quarter (190.6 vs 189.4 cm; *P* = .01) and overhand pitchers (190.6 vs 189.1 cm; *P* = .004). Conversely, sidearm pitchers had lower BMI than both three-quarter (26.6 vs 27.1; *P* = .03) and overhand pitchers (26.6 vs 27.1; *P* = .04). Sidearm pitchers also started fewer games than both three-quarter (26.5% vs 36.2%; *P* = .003) and overhand pitchers (26.5% vs 34.4%; *P* = .03). Age, weight, handedness, and total pitches thrown did not differ significantly across groups (Table 1).

**Table 1:** Demographics and Performance*^*a*^*

	Sidearm (N = 255)	Three-Quarters (n = 373)	Overhand (n = 299)	*P^*b*^*
Demographics				
Arm slot (°)	25.3 ± 6.9	39.0 ± 3.2	50.5 ± 4.9	**<.001**, **<.001**, **<.001**
Age	28.3 ± 3.5	28.3 ± 3.5	28.4 ± 3.6	.97, .92, .99
Height, cm	190.6 ± 5.3	189.4 ± 5.3	189.1 ± 5.1	**.01**, .83, **.004**
Weight, kg	96.6 ± 10.4	97.3 ± 9.9	97.0 ± 8.9	.70, .92, .91
BMI, kg/m^2^	26.6 ± 2.7	27.1 ± 2.5	27.1 ± 2.3	**.03**, ≥.99, .04
Games started, %	26.5 ± 36.1	36.2 ± 36.8	34.4 ± 35.7	**.003**, .79, **.03**
Right-handed, %	73.3	74.8	71.6	.91, .62, .89
Total pitches	3777.0 ± 3243.0	4197.0 ± 3385.0	4180.0 ± 3299.0	.27, >.99, .33
Performance				
Era	4.2 ± 1.1	4.4 ± 1.2	4.3 ± 1.3	.22, .93, .42
xFIP	4.2 ± 0.6	4.3 ± 0.6	4.3 ± 0.7	**.003**, .99, **.008**
Stuff+	102.2 ± 8.4	99.3 ± 7.8	98.9 ± 8.4	**<.001**, .82, **<.001**
Location+	99.0 ± 5.3	99.0 ± 5.5	99.5 ± 5.6	>.99, .46, .46
Pitching+	100.2 ± 8.1	98.6 ± 7.5	99.0 ± 7.9	**.04**, .83, .17
K%	23.3 ± 4.6	22.5 ± 4.1	22.7 ± 4.6	.09, .77, .35
BB%	8.4 ± 2.3	8.6 ± 2.4	8.7 ± 2.3	.60, .99,.53
Hard-hit%	35.2 ± 4.4	36.4 ± 4.1	36.3 ± 4.1	**<.001**, .83, **.006**
Swing-miss%	26.5 ± 4.6	26.0 ± 4.2	26.5 ± 4.4	.29, .28, ≥.99

a Data are presented as mean ± SD unless otherwise indicated. Pitchers were grouped by arm slot: sidearm (<33.5°), three-quarters (33.5°-44.4°), and overhand (>44.4°). BB%, walk percentage; BMI, body mass index; ERA, earned run average; K%, strikeout percentage; location+, pitch location quality metric indexed to league average (=100); pitching+, composite pitching metric indexed to league average (=100); stuff+, pitch quality metric indexed to league average (=100); swing-miss%, swinging-strike rate; xFIP, expected fielding independent pitching.

b *P* values from Tukey honestly significant difference post hoc comparisons, each row: sidearm vs three-quarters, three-quarters vs overhand, and sidearm vs overhand. Bold *P* values indicate statistical significance at <.05.

Among performance metrics, sidearm pitchers demonstrated significantly higher Stuff+ than both three-quarter (102.2 vs 99.3; *P* < .001) and overhand pitchers (102.2 vs 98.9; *P* < .001). They also had lower xFIP than both three-quarter (4.17 vs 4.34; *P* = .003) and overhand pitchers (4.17 vs 4.33; *P* = .008), as well as lower hard-hit percentage than both three-quarter (35.2% vs 36.4%; *P* < .001) and overhand pitchers (35.2% vs 36.3%; *P* = .006). Pitching+ was higher in sidearm compared with three-quarter pitchers (100.2 vs 98.6; *P* = .04), with no significant difference between sidearm and overhand pitchers. No significant differences were observed across groups for ERA, location+, strikeout rate, walk rate, or swing-and-miss percentage (Table 1).

### Pitch Characteristics

Pitch usage differed by arm slot (Table 2). Sidearm pitchers relied more heavily on sinkers (23.7%) and less on 4-seam fastballs (27.3%) than both three-quarter (16.7% and 34.7%) and overhand pitchers (11.2% and 38.4%; all *P*≤ .02). Sweeper usage was also highest among sidearm pitchers (18.8%), exceeding both three-quarter (11.8%; *P* < .001) and overhand pitchers (8.1%; *P* < .001). Cutter usage was higher in overhand than sidearm pitchers (16.1% vs 11.5%; *P* = .007), while slider usage was modestly higher in sidearm than three-quarter pitchers (21.8% vs 18.4%; *P* = .02). Pitch-level characteristics were otherwise largely similar across groups. Sidearm pitchers showed lower slider velocity and higher slider spin than both three-quarter and overhand pitchers, while fastball velocities and spin rates did not differ significantly across arm slot groups (Table 2).

**Table 2:** Pitch Characteristics*^*a*^*

	Sidearm (n = 255)	Three-Quarters (n = 373)	Overhand (n = 299)	*P^*b*^*
Changeup				
Percentage	12.0 ± 11.2	12.4 ± 10.0	12.0 ± 10.2	.92, .92, ≥.99
Velocity	86.4 ± 3.0	86.4 ± 2.7	85.7 ± 3.1	.99, **.01**, **.03**
Spin	1806.0 ± 252.0	1763.0 ± 247.0	1702.0 ± 260.0	.12, .01, **<.001**
Curveball				
Percentage	11.0 ± 11.4	9.4 ± 8.5	11.5 ± 8.6	.29, **.04**, .88
Velocity	79.3 ± 3.0	78.8 ± 3.3	79.0 ± 3.3	.39, .78, .73
Spin	2507.0 ± 282.0	2482.0 ± 281.0	2480.0 ± 262.0	.70, >.99, .66
Cutter				
Percentage	11.5 ± 12.1	12.5 ± 11.8	16.1 ± 16.2	.75, **.03**, **.007**
Velocity	88.6 ± 2.8	89.1 ± 2.4	89.3 ± 2.6	.12, .91, .07
Spin	2350.0 ± 182.0	2360.0 ± 193.0	2356.0 ± 179.0	.87, .98, .95
Four-Seam				
Percentage	27.3 ± 19.1	34.7 ± 16.7	38.4 ± 16.7	**<.001**, **.02**, **<.001**
Velocity	93.7 ± 2.5	94.1 ± 2.2	93.8 ± 2.3	.11, .16, .96
Spin	2272.0 ± 155.0	2274.0 ± 146.0	2262.0 ± 137.0	.99, .56, .71
Sinker				
Percentage	23.7 ± 18.3	16.7 ± 16.3	11.2 ± 14.3	**<.001**, **<.001**, **<.001**
Velocity	93.3 ± 2.6	93.6 ± 2.3	93.4 ± 2.5	.23, .60, .82
Spin	2183.0 ± 157.0	2182.0 ± 176.0	2179.0 ± 173.0	>.99, .97, .97
Slider				
Percentage	21.8 ± 16.9	18.4 ± 13.0	20.6 ± 15.2	**.02**, .16, .63
Velocity	84.6 ± 3.0	85.2 ± 2.6	85.3 ± 2.6	**.04**, .84, **.01**
Spin	2445.0 ± 223.0	2399.0 ± 226.0	2379.0 ± 192.0	**.044**, .52, **.003**
Sweeper				
Percentage	18.8 ± 13.3	11.8 ± 11.5	8.1 ± 7.4	**<.001**, **.03**, **<.001**
Velocity	81.6 ± 2.5	82.1 ± 2.6	82.3 ± 2.3	.20, .76, .07
Spin	2545.0 ± 222.0	2528.0 ± 223.0	2525.0 ± 188.0	.79, >.99, .78

a Data are presented as mean ± SD. Pitch usage percentage, velocity (mph), and spin rate (rpm) for 7 pitch types stratified by arm slot: sidearm (<33.5°), three-quarters (33.5°-44.4°), and overhand (>44.4°).

b *P* values from Tukey honestly significant difference post hoc comparisons, each row: sidearm vs three-quarters, three-quarters vs overhand, sidearm vs overhand. Bold *P* values indicate statistical significance at <.05.

### Performance Metrics

**Figure 2.**
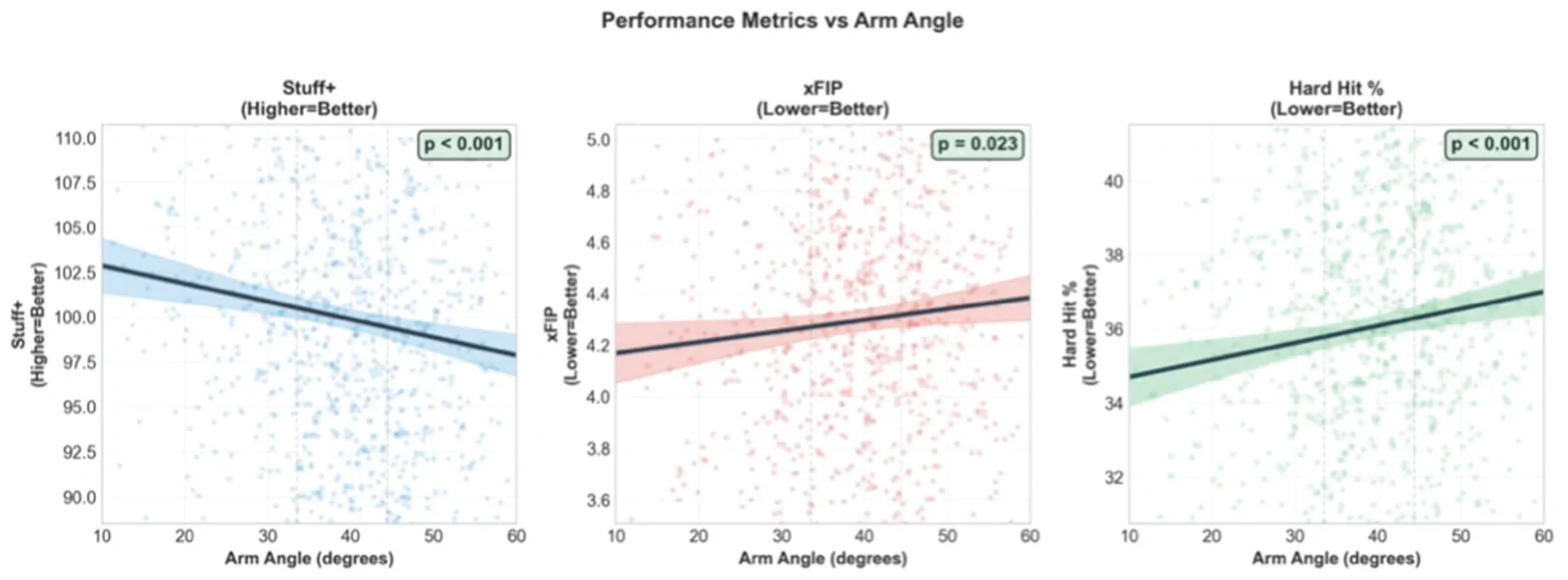
Association between arm slot and performance metrics. Each point represents 1 pitcher, with mean arm slot across the study period plotted against the corresponding aggregated performance metric. Linear regression lines with 95% CIs depict these relationships. Dashed vertical lines indicate arm slot boundaries for the sidearm (<33.5°), three-quarter (33.5°-44.4°), and overhand (>44.4°) groups. xFIP, expected fielding independent pitching.

Linear regression analyses examining the continuous association between arm slot and performance metrics are presented in Table 3, with relationship curves shown in Figure 2. Arm slot was standardized to 1 SD (10.9°), such that coefficients represented the change in each metric per 1-SD increase toward a more overhand delivery. A more overhand arm slot was associated with lower Stuff+ (β = −1.09; *P* < .001), higher xFIP (β = 0.05; *P* = .02), and higher hard-hit percentage (β = 0.50; *P* < .001), indicating a less favorable performance profile than a more sidearm delivery (Table 3).

**Table 3:** Association Between Arm Slot and Performance Metrics*^*a*^*

Metric	β per SD (95% CI)	*P*
Stuff+	–1.09 (–1.62 to –0.56)	**<.001**
xFIP	0.05 (0.01 to 0.09)	**.02**
Hard-hit%	0.50 (0.23 to 0.77)	**<.001**

a Association between arm slot and performance metrics. Linear regression with arm slot standardized to 1 SD (10.9°). β represents the change in each metric per 1-SD increase in arm slot. Bold *P* values indicate statistical significance at *P* < .05. Stuff+, pitch quality metric indexed to league average (=100); xFIP, expected fielding independent pitching.

#### Injury Burden

**Figure 3.**
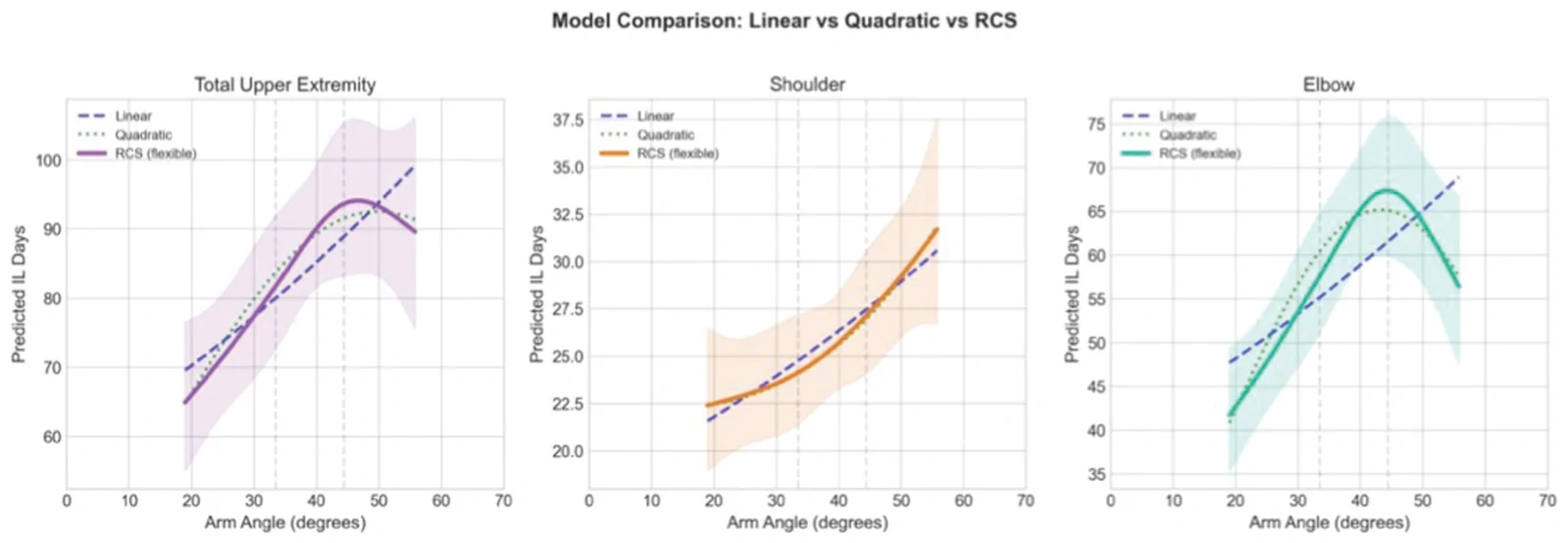
Predicted injury burden by arm slot. Predicted injured list (IL) days from negative binomial models at median exposure (median total pitches). Curves depict linear, quadratic, and restricted cubic spline (RCS) model fits; shaded regions indicate 95% CIs. Vertical dashed lines denote arm slot group boundaries.

Figure 3 displays predicted IL days across the arm slot spectrum using linear, quadratic, and restricted cubic spline model fits. All models were adjusted for total pitches thrown and percentage of games started. Based on formal model comparisons and quadratic term testing, quadratic models provided superior fit for combined upper extremity (*P* = .045) and elbow (*P* < .001) outcomes, while shoulder injury was best characterized by a linear model. These model specifications were used for all subsequent analyses.

Univariable negative binomial regression results are presented in Table 4. More overhand arm slots were significantly associated with greater injury burden across all 3 outcomes. Each 1-SD (10.9°) shift toward a more overhand delivery was associated with 9% more combined shoulder and elbow IL days (IRR = 1.09; *P* = .01), 11% more shoulder IL days (IRR = 1.11; *P* = .002), and 8% more elbow IL days (IRR = 1.08; *P* = .03). Peak injury burden occurred at 50° for combined upper extremity and 45° for elbow (Table 4).

**Table 4:** Association Between Arm Slot and Injury Burden*^*a*^*

Outcome	IRR (95% CI)	*P*	Model	Peak
Combined (S + E)	1.09 (1.02-1.17)	**.01**	Quadratic*^*b*^*	50°
Shoulder	1.11 (1.04-1.18)	**.002**	Linear	–
Elbow	1.08 (1.01-1.15)	**.03**	Quadratic*^*b*^*	45°

a Negative binomial generalized linear model with log(total pitches) and percentage of games started included as covariates. Bold *P* values indicate significance at <.05. Dash indicates not applicable for the linear model. CI, confidence interval; IRR, incidence rate ratio per 1-SD (10.9°) increase in arm slot; S + E, combined shoulder and elbow.

b Quadratic model; peak represents arm slot with highest injury burden calculated as –β_1_/(2β_2_).

The primary analysis tested whether arm slot remained associated with injury burden after adjusting for established risk factors: Stuff+ and 4-seam fastball velocity (Table 5). Arm slot remained a significant independent predictor across all outcomes. Each 1-SD shift toward a more overhand delivery was associated with 13% more combined shoulder and elbow IL days (IRR = 1.13; *P* < .001), 14% more shoulder IL days (IRR = 1.14; *P* < .001), and 12% more elbow IL days (IRR = 1.12; *P* < .001). In multivariable models, peak injury burden occurred at 57° for combined upper extremity and 46° for elbow.

**Table 5:** Multivariable Models: Independent Effect of Arm Slot on Injury Burden*^*a*^*

Outcome	Arm Slot IRR (95% CI)	*P*	Model	Peak	Stuff+ IRR/FF Velocity IRR	*P*
Combined (S + E)	1.13 (1.06-1.21)	**<.001**	Quadratic*^*b*^*	57°	S: 1.19 V: 1.23	S: **<.001** V: **<.001**
Shoulder	1.14 (1.06-1.22)	**<.001**	Linear	–	S: 1.19 V: 1.10	S: **<.001** V: **.02**
Elbow	1.12 (1.05-1.20)	**<.001**	Quadratic*^*b*^*	46°	S: 1.19 V: 1.29	S: **<.001** V: **<.001**

a Negative binomial generalized linear model adjusted for log(total pitches), percentage of games started, Stuff+, and 4-seam velocity. Tests whether arm slot has an independent effect beyond pitch quality. Bold *P* values indicate *P* < .05. Dash indicates not applicable for the linear model. CI, confidence interval; IRR, incidence rate ratio per 1-SD (10.9°) increase in arm slot; S, stuff+; S + E, combined shoulder and elbow; stuff+, pitch quality metric indexed to league average (=100); V, velocity; FF, four-seam fastball.

b Quadratic model; peak calculated as –β_1_/(2β_2_).

## Discussion

The principal finding of this study was that sidearm deliveries were associated with lower upper-extremity injury burden while maintaining a favorable performance profile. Pitchers with sidearm slots maintained comparable fastball velocities across arm slot groups and demonstrated higher composite Stuff+, more favorable xFIP, and lower hard-hit rates. These outcomes were accompanied by distinct pitch usage patterns across the arm slot spectrum: compared with overhand and three-quarter pitchers, those with sidearm deliveries threw fewer 4-seam fastballs and cutters and relied more heavily on sinkers and sweepers. Shoulder injury burden increased linearly with more overhand arm slots, whereas elbow and total upper-extremity burden followed a nonlinear relationship, peaking in the overhand range (45°-57°). Notably, these associations persisted in multivariable models controlling for workload, velocity, and pitch quality, traits repeatedly linked to injury risk in modern pitching, supporting arm slot as a clinically relevant feature associated with both durability and performance.

The elbow findings in this study challenge the prevailing recommendation that a three-quarter arm slot is the safest. Much of the prior literature supporting this recommendation is based on laboratory motion capture studies that quantified joint kinetics during controlled throwing assessments, whereas the present study examined cumulative, real-world time-loss injury burden across a large MLB cohort. Prior results have been mixed across populations. Albright et al^4^ noted higher elbow pain severity in sidearm throwers, and Okoroha et al^36^ found increased medial elbow torques in youth sidearm pitchers. Similarly, Aguinaldo and Chambers^3^ suggested that sidearm pitching in adult pitchers is associated with higher elbow valgus loading. In contrast, a substantial body of literature across pitcher levels has suggested that more overhand deliveries are associated with greater elbow loading.^9,12,25,33,37,42^ Our results support the general direction that elbow burden rises as arm slot shifts toward more overhand deliveries, but highlight that the pattern is not monotonic. The modeled peak in elbow injury burden occurred at 45° to 46°, near the threshold between three-quarter and overhand deliveries and within the lower end of the overhand range, rather than continuing to rise at more overhand arm slots. This helps contextualize the findings of Escamilla et al^14^ in a separate cohort of professional pitchers, where the three-quarter group exhibited the highest elbow kinetics. While our data place the peak just within the overhand range, Escamilla et al^14^ may have observed a similar nonlinear pattern, with lower values in sidearm deliveries and the highest elbow kinetics occurring near the transition toward overhand slots.

Shoulder injury burden increased in a linear fashion as arm slot became more overhand, which generally aligns with prior motion capture work in higher level pitchers. Across cohorts, more overhand slots have been associated with greater shoulder loading, including higher peak shoulder internal rotation torque and shoulder anterior forces,^13,25,37,42^ along with less favorable shoulder torque efficiency.^12^ In contrast, multiple motion capture studies have reported higher elbow flexion torque in sidearm slots, which has been proposed to increase loading of the proximal biceps tendon and superior labrum, and has led investigators to recommend a three-quarter slot as a compromise between competing shoulder stresses.^13,25^ Although the present study did not evaluate specific diagnoses such as SLAP tears, the consistent linear relationship between more overhand arm slots and greater shoulder time loss suggests that, at a population level, sidearm deliveries are associated with a lower overall shoulder injury burden even if specific shoulder pathologies may follow different slot-dependent mechanisms. Together with the nonlinear elbow findings, this suggests a redistribution of joint loading across the arm slot spectrum. The lower end of the overhand range, near the transition from three-quarter to overhand delivery, may represent a mechanical zone in which elbow valgus stress remains elevated before load shifts proximally. At more extreme overhand slots, greater shoulder abduction and a more vertical force direction may transfer stress toward the shoulder, explaining why shoulder burden continues to rise while elbow burden declines beyond its peak.

Arm slot should be interpreted as a composite biomechanical outcome rather than a marker of any single joint position. At ball release, arm slot reflects coordinated contributions from lateral trunk flexion, shoulder abduction, and elbow positioning, which pitchers combine to produce a given delivery style.^12,25^ Although the present data set did not permit these individual components to be disaggregated, prior work suggests that variation in arm slot is largely driven by proximal mechanics (particularly trunk motion and sequencing) rather than isolated distal joint behavior.^12,25^ Within that framework, the combination of equivalent fastball velocity and lower elbow injury burden in sidearm pitchers may reflect greater mechanical efficiency of these delivery styles. Manzi et al^25^ reported that professional pitchers with more sidearm slots demonstrated delayed trunk rotation timing, which may improve proximal-to-distal energy transfer, and Dong et al^12^ similarly found greater elbow and shoulder torque efficiency in sidearm strategies among elite collegiate pitchers. Together, these findings suggest that as strength, motor control, and trunk coordination mature, some pitchers may adopt more sidearm deliveries that allow comparable velocity to be generated with less distal joint stress.

This interpretation may also help contextualize Manzi et al’s^25^ observation that high school pitchers more often used overhand deliveries than their taller professional counterparts. In the present cohort, sidearm pitchers were also the tallest group, exceeding both three-quarter and overhand pitchers by approximately 1.5 cm. Taken together, these findings raise the possibility that body habitus may partly influence habitual arm slot, with taller pitchers potentially better positioned to achieve more sidearm deliveries through differences in trunk orientation, leverage, or both. Whether this relationship is mechanistic or instead reflects selection and developmental pressures remains uncertain.

Sidearm pitchers demonstrated higher Stuff+, lower hard-hit rates, and more favorable xFIP compared with more overhand deliveries. These differences were not explained by fastball velocity or spin, which did not differ significantly across arm slot groups. Instead, sidearm pitchers showed a clear repertoire shift away from 4-seam fastballs and cutters toward greater reliance on sinkers and sweepers. These pitches create different movement patterns and approach slots at the plate, and their increased usage likely contributes to the performance differences observed. From an injury standpoint, fastballs have consistently been identified as high-stress pitches,^21^ although it remains unclear whether shoulder or elbow loading differs meaningfully between 4-seam and sinker fastballs. While changeups have been proposed as lower-stress offerings due to pronation-dominant mechanics,^17,19^ no differences in changeup usage were observed across arm slot groups. Taken together, the slot-dependent repertoire patterns observed here may represent one pathway linking arm slot, pitch selection, performance, and cumulative injury burden.

Sidearm pitchers also started fewer games than three-quarter and overhand pitchers, indicating that this delivery style was more common among relievers in the present cohort. This may partly explain why sidearm pitchers demonstrated favorable pitch quality and contact management metrics despite starting less frequently, as relievers often operate in shorter outings that emphasize specialized arsenals and matchup advantages. However, injury models adjusted for both total pitches thrown and percentage of games started, suggesting that the association between arm slot and injury burden was not explained solely by role. Pitch selection was not included in the primary multivariable models because repertoire may be partly determined by arm slot rather than acting solely as an independent confounder; therefore, adjusting for pitch usage could overcorrect by removing part of the pathway through which arm slot influences injury risk.

Unlike prior MLB investigations that relied on horizontal release position as a surrogate for arm slot, the present study leverages MLB's recently available, directly measured arm slot metric derived from the Hawk-Eye optical tracking system. Horizontal release position, defined as the lateral distance of ball release from the center of the pitching rubber, has been used to approximate delivery style, with more central release points interpreted as reflecting a more overhand slot and release points farther from the rubber's center interpreted as reflecting a more sidearm slot. Findings based on this proxy, however, have been inconsistent: Portney et al^39^ associated release points farther from center with increased UCL injury risk, implicating more sidearm deliveries, whereas others linked more central release points to greater UCL risk,^31,44^ and several reported no association.^26,29^ These discrepancies likely reflect the limitations of release-position surrogates, which are influenced by stride length, mound positioning, and pitcher anthropometrics rather than true arm orientation. Efforts to estimate arm position from broadcast video have similarly yielded null findings. For example, Lipa et al^22^ reported no differences in arm or elbow angles between UCL cases and controls, though interpretation was constrained by variable camera geometry across stadiums and manual angle estimation. By directly quantifying arm slot and linking it to longitudinal injury burden across multiple MLB seasons, the current analysis provides a more rigorous framework for determining which arm slots track with shoulder and elbow durability in professional pitchers.

From a clinical and player development standpoint, these findings indicate that sidearm slots in elite pitchers can both have a lower injury risk and be effective. Arm slot, however, is tightly coupled with trunk orientation and sequencing, such that any meaningful change in slot is unlikely to occur through isolated cueing and instead would require coordinated, whole-body mechanical adaptation. At the same time, the present study does not address whether arm slot is readily modifiable within an individual pitcher. Although there are examples in professional player development of pitchers adopting more sidearm slots with subsequent performance gains,^1^ whether deliberate slot modification can reliably preserve effectiveness while reducing injury burden remains unknown. Prospective, targeted work is needed to define the feasibility and limits of arm slot adjustment and to determine whether a pitcher's habitual slot represents a stable biological and mechanical optimum or a modifiable characteristic. By linking arm slot to longitudinal time-loss injury burden rather than isolated diagnoses or single-event kinetics, these findings support a more clinically relevant durability framework that maps directly to availability and real-world cost to the athlete and the team.

## Limitations

This study has several important limitations. Injury outcomes were derived from publicly reported IL data, which may be subject to misclassification, incomplete diagnostic detail, or underascertainment if players continued to pitch through injury without formal IL placement. IL-based capture may also miss offseason injuries, potentially underestimating total injury burden. Additionally, shoulder and elbow injuries were analyzed both jointly and as broad anatomic categories rather than as diagnosis-specific entities. While this approach may obscure slot-specific associations with pathologies, it allowed assessment of the full spectrum of upper-extremity time loss attributable to pitching mechanics. Further, arm slot is a composite kinematic descriptor and does not isolate the individual contributions of shoulder abduction, elbow flexion, or trunk tilt; therefore, these data cannot distinguish different joint-level movement strategies that yield similar arm slot values. Submarine pitchers were excluded because of small sample size, and no inferences can be made regarding injury burden or performance in that delivery group. Additionally, this cohort was limited to MLB pitchers, and the extent to which these associations generalize to younger, developing athletes remains uncertain.

## Conclusion

In this MLB cohort, sidearm deliveries were associated with lower shoulder/elbow time-loss burden, superior pitch quality, and lower hard-hit rates. Shoulder injury burden increased linearly with more overhand deliveries, whereas elbow burden followed a nonlinear pattern that peaked within the overhand range. These associations were independent of workload, velocity, and pitch quality. Arm slot may represent a clinically relevant marker of pitcher injury risk that warrants prospective study.
